# Stakeholders’ Response to IFRS adoption/ convergence on accounting quality and disclosures: A bibliometric review of Scopus database

**DOI:** 10.1016/j.heliyon.2023.e23912

**Published:** 2023-12-18

**Authors:** Sakshi Bathla, Anil K. Sharma, Vinay Kandpal

**Affiliations:** a LM Thapar School of Management Derabassi Campus, Chandigarh, Punjab, India; b Department of Management Studies, Indian Institute of Technology Roorkee, Uttarakhand, India; c Department of Management Studies, Graphic Era Deemed to be University, Dehradun, Uttarakhand, India

**Keywords:** IFRS, Stakeholders, Perceptions, Bibliometric review

## Abstract

This study characterises the results of behavioral accounting research on International Financial Reporting Standards (IFRS) adoption published in various journals. It: (a) provides an integrated overview of the extant literature available on the Scopus database, (b) locates their contributions, (c) identifies knowledge gaps and (d) derives a unique hypothesis for future investigation. This review presents an analysis of the studies on IFRS adoption/convergence considering the response of various stakeholders to IFRS adoption on issues including *accounting quality* and *disclosure requirements*. The present paper analyses 106 articles published between 2005 and 2021. Preparers (accountants) and users including academicians, researchers, policymakers, and regulatory and standard-setting bodies such as IASB may use this examination as a guideline to conduct further inspections into the standard-setting processes and the related issues.

## 1 Introduction

Globalization has created an unavoidable demand for convergence of financial reporting that is being met by International Financial Reporting Standards (IFRS). These international accounting standards have brought significant changes in accounting procedures and financial reporting practices worldwide. Specifically, these regulatory changes have led to substantial modifications in the preparation of financial statements and the presentation of financial information to various stakeholders globally. It has defined them as an innovation of historical proportions. IFRS have attracted rumbles amongst the accounting community since the establishment of the International Accounting Standards Committee (IASC) in 1973 by the bigwigs viz., AICPA (American Institute of Certified Public Accountants), ICAEW (Institute of Chartered Accountants of England and Wales) and CICA (Canadian Institute of Chartered Accountants) [1]. Since then, this area has progressed exponentially, offering various insights to stakeholders.

Countries around the world are adopting IFRS. Despite uncertainty about the impact of IFRS adoption, the efforts paid off with a public commitment from 166 countries to adopt the global movement. Out of these countries, more than 144 countries already adopted IFRS (www.ifrs.org). For this purpose, the International Accounting Standards Board (IASB) has divided the globe into five major regions: Europe, Africa, the Middle East, Asia-Oceania, and the Americas. Many researchers have conducted numerous studies across five areas accounting for various jurisdictions. Given the importance of internationalization of the standards, the area has attracted attention from all over the world. The early scholarly papers, including some literature reviews dealing with IFRS convergence, have recognized the need for international accounting standards and highlighted the need to reconcile regional GAAPs with global accounting standards. Unfortunately, no concentrated, thorough, and systematic attempt has been made to classify literature on stakeholders' participation to capture their responses on the IFRS adoption, accounting quality and disclosure requirements. The need for and importance of such work have been highlighted by many studies, including [[2], [3], [4], [5], [6]]. [7] reviewed papers published from 2005 to 2014. Apart from these, our paper identified various domains, including the impact and financial market actors’ perceptions.

In an early work [8], highlighted the use of participant perceptions to manage large-scale organizational change and suggested it as a way to identify the key issues in planned change efforts. Similarly [9], considering the theory of institutional isomorphism, drew parlance between the literature on organizational change that continually accepts the usefulness of understanding how the individual participants perceive the effects of change in progress and the fundamental differences in financial reporting systems. At the same time [10], highlighted the importance of the perceptional approach as a source of further development. Thus, the research investigates stakeholders’ perceptions and knowledge on various issues, including the costs, benefits and applicability of IFRS, as it seems scarce and scattered, which motivates us to write this paper.

The current study offers invaluable comprehension into the status of research in the area of IFRS, considering stakeholders’ perceptions. It intends to provide systematic information on year-wise trend analysis, an elaborate study on authors and yearly citation metrics, sources along with annual citation metrics of these avenues, relevant keywords and their usage depicting the direction of publication progression over the years, followed by the contributions by various countries to literature and thus highlighting countries that need to be a focus for future research. The paper seeks to fill the vacuum by conducting a systematic review of 106 papers published from 2005 to 2021. 2005 marked the simultaneous adoption of IFRS by more than 7000 companies in 25 European countries [11]. Apart from a wider readership and a higher impact factor, the current platform is selected as it publishes valuable research in broader areas of business and economics. Moreover, it has posted enough evidence on many contemporary themes about accounting, such as accounting education, optimization to financial accounting, financial statement comparability, factors affecting IFRS adoption, governance mechanism on IFRS adoption, etc., which provides us confidence in the platform. The observations provided in this study would provide direct valuable insights and introduce a few avenues for future research. To the best of our knowledge, such a concentrated, thorough and systematic endeavour has yet to be undertaken, emphasizing the pioneering contribution of the current work to the body of IFRS literature.

**Fig. 1:**
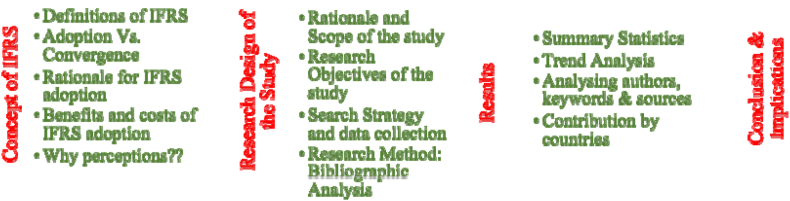
Structure of the research.

The flow of this research article is shown in Fig. 1. Section 2 describes the basic concept of IFRS and the role of perceptions. Section 3 illustrates the research design of the study which briefs the rationale, scope, objectives, search strategy and methodology adopted for this study. Section 4 provides the bibliometric analysis as the results derived from this study. Section 5 provides the limitations and recommendations for future research. Section 6 provides the policy issues and implications of this research. Lastly, the conclusion in Section 7. The observations provided in this study direct towards valuable insights and introduce to few avenues for future research. To the best of our knowledge, such a concentrated, thorough and systematic endeavour has not been undertaken which emphasizes the pioneering contribution of the current work to the body of IFRS literature.

**Fig. 2:**
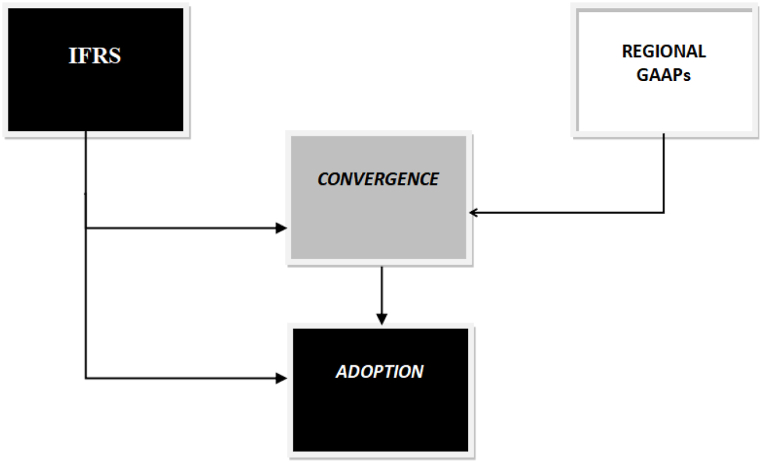
Adoption vs. Convergence, (Source: Authors).

## 2 The concept of IFRS

Various attempts have been made to define IFRS by various individuals and organizations. This section provides various definitions of IFRS furnished by the apex regulatory bodies and academicians.

### 2.1 Definition of IFRS

The first and the most comprehensive attempt to define IFRS was made by the paramount International Accounting Standards Board (IASB) to define IFRS “as standards that constitute a globally recognized set of standards for the preparation of financial statements by business entities. IFRS prescribes:

• The items that should be recognized as assets, liabilities, income, and expenses.
• How to measure those items.
• How to present them in a set of financial statements; and
• Related disclosures about those items.”

Such that they aim to be applied for financial reporting by publicly listed companies worldwide [1], these standards thus comprise International Accounting Standards (IAS) which were issued before 2001; Standing Interpretations Committee (SIC) which were issued before 2001; International Financial Reporting Standards (IFRS) - issued after 2001; Interpretations originated from International Financial Reporting Interpretations Committee (IFRIC) - issued after 2001. So, there are 28 IAS, 8 SIC, 17 IFRS and 17 IFRIC. In another attempt, the American Institute of Certified Public Accountants (AICPA) defined International Financial Reporting Standards (IFRS) as “a set of accounting standards developed by the International Accounting Standards Board (IASB) that is becoming the global standard for the preparation of public company financial statements.”

So, we define IFRS as *“a single set of accounting rules established by IASB for financial reporting by various business organizations across the* globe*.”*

### 2.2 Adoption vs. convergence/methods of IFRS implementation

Countries willing to adopt IFRS have two methods to do so: Direct and Indirect method. The direct method also called as *adoption* involves the implementation of IFRS as they are, in a particular jurisdiction. As defined by Ref. [12], “adoption of IFRS means the use of International Financial Reporting Standards as primary GAAP by domestically listed and unlisted companies in their consolidated financial statements for external financial reporting. This means the basis of the presentation note and the auditor's report indicates that financial statements are prepared according to IFRSs”. However, the indirect method also called as the *convergence* involves the implementation of IFRS with a few ‘informed divergences’ [13]. defines “convergence as a means to achieve harmony with IFRS or to design and maintain national standards in a way that financial statements are prepared by national accounting standards that draw an unreserved statement of compliance with IFRS.” Convergence, though, is a cut-short strategy for a jurisdiction that may facilitate adoption over the transitional period. Convergence, however, doesn't substitute adoption. So, regardless of the different pathways towards IFRS, the result should be full adoption of IFRS Standards as issued by IASB [14]. So, convergence means a way to align indigenous standards with international standards with the ultimate goal of fully adopting the requirements of the international standards. Fig. 2 below depicts the meaning and way towards convergence and adoption.

### 2.3 Rationale for IFRS

The IASB focuses on transparency, accountability and efficiency as the rationale for the IFRS adoption. *Transparency* enhances the quality of information and international comparability to help participants make informed decisions. *Accountability* aims to reduce the information gap between the savers and users of the capital. *Efficiency* improves by using a single trusted accounting language that identifies opportunities and threats across the globe and thus improves capital allocation. These rationales have been identified by many researchers including [1,[15], [16], [17], [18], [19], [20]].

### 2.4 Benefits and costs of IFRS

Widespread implementation of IFRS offers the stakeholders numerous advantages as well as poses disadvantages. On one hand, it offers benefits like Transparency, Efficiency [18], Accountability, higher value relevance & and more timely recognition of losses [17], rise in trading volumes [21] and a decrease in the earnings management [22]. However, on the other hand, it may pose disadvantages such as a rise in price uncertainty [21] negative net equity [23], future research and debate [24], different legal orientations [25], Language [26] and IFRS education [27].

### 2.5 IFRS vs. GAAP

While GAAP and IFRS share many similarities, there are several contrasts, beyond the regions in which they're applied. The text below lists the differences between the converged I-GAAP (now Ind-AS) and IFRS. While Ind-AS is developed by the Ministry of Corporate Affairs (MCA) keeping India's business interests in consideration, IFRS are developed by the International Accounting Standards Board [28]. Under IFRS, major financial statements comprise (a) a Statement of financial position (b) a Statement of profit and loss (c) a Statement of changes in equity for the period (d) a Statement of cash flows for the period. Whereas major statements under Ind-AS comprise (a) a Balance Sheet (b) a Profit and loss account (c) a Cash flow statement (d) a Statement of changes in equity (e) Notes to financial statements (f) Disclosure of accounting policies. Most of the erstwhile GAAPs are historical cost concept-based, whereas IFRS preaches the Fair Valuation [29]. Moreover, most of the indigenous GAAPs are rules-based, whereas IFRS allows room for discretion and is flexible in many aspects [30]. The primary distinction between U.S. GAAP and IFRS is in their respective levels of predictability. Nevertheless, the investors do not seem to completely recognize the disparity in predictability between U.S. Generally Accepted Accounting Principles (GAAP) and International Financial Reporting Standards (IFRS), as there are no major and consistent discrepancies observed in the value-relevance features of these two standards [31].

### 2.6 Why perceptions???

[32] defined perceptions as “a sensory experience in which an individual observes a behaviour, event, or condition; forms interpretations of the factors observed; develops attitudes; and allows the processed observation to become a factor influencing his or her behaviour” [33]. has defined it as “a process which involves the recognition and interpretation of stimuli which register on our senses”. So by and large, since perceptions are not necessarily a reality as they are not always accurate or correct, it is important to investigate how and in which direction they may affect a new change. Hence, further research is sought to validate if these perceptions are correct [3]. The perceptional approach has been referred to by Ref. [34] as the interpretative approach of “research that reflects an investigation of the status quo from individuals’ perspectives, investigating their opinions, seeking their points of view to create higher order categories and reflections that summarize the shared reality of participants”.

## 3 Research design of the study

The current study reflects the importance of the perceptions of various stakeholders and covers a variety of themes that were highlighted through this analysis. The study accomplishes three major objectives in this study. We have adopted bibliometric analysis as a methodology to achieve the stated objectives. Bibliometric techniques offer more quantitative rigour in comparison to a narrative literature review [35].

### 3.1 Rationale and scope of the study

Globalization is the root cause of the internationalization of accounting standards. Countries adopting IFRS are more exposed to the benefits of globalization of businesses. More and more national accounting regulatory bodies are in favour of adopting IFRS, however, individuals’ participation in the standards-setting process is less than expected. Before finalizing any standard at any level, the perceptions and viewpoints of stakeholders in the field must be gauged to make it acceptable and implementable at all levels. Very few studies focus on the participation and perception of ultimate preparers and users of accounting information in decision-making. Considering the above-mentioned scenario, the current analyses and presents various issues about IFRS adoption at one platform in a structured manner. The key stakeholders within the accounting profession encompass auditors, investors, managers, regulators, and standard setters. Through an examination of stakeholders' reactions to the adoption or convergence of International Financial Reporting Standards (IFRS), valuable insights can be obtained into the perception of the worldwide transition to IFRS and the anticipated advantages and obstacles identified by these stakeholders. The adoption and convergence of International Financial Reporting Standards (IFRS) plays a crucial role in the process of globalization accounting standards. Understanding the reactions of stakeholders can provide valuable insights for making informed decisions about the dissemination, modification, or interpretation of these standards.

Through a comprehensive analysis of the extant body of literature, it is possible to discern deficiencies or gaps in the existing knowledge base. This information can serve as a valuable resource for future researchers, providing guidance on new areas of study that have the potential to make substantial contributions to the current body of knowledge. Organizations, particularly those contemplating the adoption of International Financial Reporting Standards (IFRS) or those already implementing IFRS, can acquire valuable knowledge regarding optimal strategies, obstacles encountered by peers, and potential benefits of these standards by examining stakeholders' responses.

### 3.2 Research objectives of the study

The research objectives of this paper are three-fold. One is to present the studies on IFRS adoption considering the perceptions of various stakeholders in an organized and easily interpretable way. The idea is to review the existing literature on IFRS perceptions and implementation. It seeks to provide an explicit view of the status of research on various issues about IFRS adoption and segregate them into various suitable categories based on authors, publication year, publication source, keywords, region of publication and author collaborations. The study thus brings different sources of information on IFRS to a common platform and highlights the important issues addressed in those sources to make it readily available to different stakeholders. Finally, to identify the research gaps and make recommendations for future research avenues in the process.

### 3.3 Search strategy and data collection

The paper examines studies on the perceptions of various stakeholders on the adoption of IFRS in their jurisdictions that have been published in reputed journals and other sources. For this purpose, we have used 106 (*n*) items. We have used the string “Perceptions AND IFRS”. In total, we found 144 papers. The search results showed papers published from 2005 to 2022. We limited our search to the Scopus database, the publication year 2021 for the reason that year 2022 was still going on at the time of writing this paper. We further limited the search criteria to subject area, document type, source type and language. Table 1 below provides detailed search criteria adopted for this study.

**Table 1:** Search criteria.

		n
Total documents		144
Limit to:		
Year	2005–2021	137
Subject area	• Business, Management and Accounting • Economics, Econometrics and Finance • Social Sciences	124
Document Type	Article	111
Source Type	Journal	108
Language	English	106
**Total papers for analysis**		**106**

**Table 2:** Descriptive summary of the findings.

Description	Results
Documents	106
Average citations per document	14.30
Sources (Journals, Books etc.)	66
Period	2005–2021
Authors	259
Documents per Author	0.409
Authors per Document	2.44
Collaboration Index	1.114

An assessment of a net of 106 outputs was undertaken after checking the relevance of *stakeholders’ perceptions* to develop various classifications of literature presented in the study. After careful analysis of the different timelines, sources, authors and various perceptional aspects of IFRS have been identified and presented in the paper at appropriate places.

### 3.4 Research Methodology: Bibliometric analysis

To accomplish the objectives of this study, we have conducted a bibliometric analysis (a) to handle a large volume of scientific data, and (b) to produce a high research impact [36]. The papers retrieved were analysed for bibliometric indicators in Biblioshiny, an R Statistical Software interface. This paper used the software to report the descriptive statistics. The software also used Bibliometrix R software for citation and co-citation analysis. This analysis tool generally provides the evolution of a concept over the years. It also reveals the emergent themes relevant to the concept. This paper thus provided relevant and emergent issues in IFRS that could be considered for further investigation. It also reveals the sources and authors that are creating value in the field. A bibliometric analysis shows various stages in a field of study and the new areas of investigation that can be studied further [37].

## 4 Results

### 4.1 Summary statistics

This section provides the analysis and findings from the present bibliometric analysis of 106 documents related to stakeholders' responses on IFRS implementation published between 2005 and 2021. Table 2 below presents the descriptive summary of the findings from this analysis. The articles published in the Scopus database during this period earned an average of 9.094 citations. A high average of citations per document shows an accelerated growth of academic articles in the area. The results also depict that 259 different authors have analysed the perceptions on IFRS implementation, who have been cited 2618 times in total. On average, 2.44 authors have contributed per document while every single author contributed to at least 0.409 documents. This summary implies the development of research in perceptions of IFRS implementation all over the world. There's a compelling collaboration index at 1.114 signifying low collaborations amongst countries. Major collaborations are coming from Australia, Europe and the USA.

### 4.2 Trend analysis

**Fig. 3:**
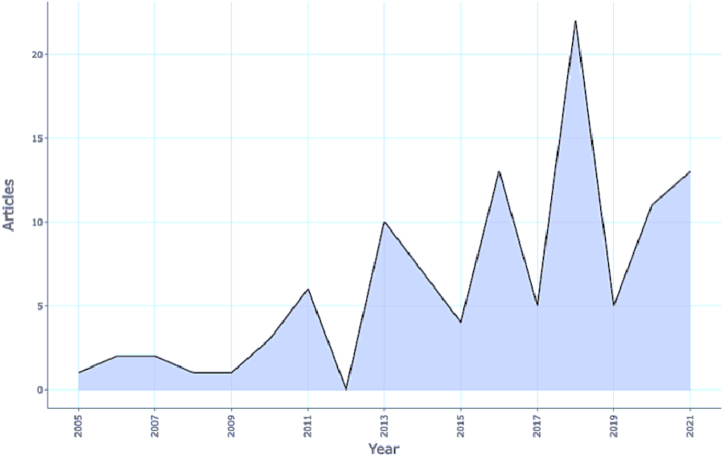
Annual scientific production of research articles.

Fig. 3 below provides the annual scientific production of research articles on stakeholders' perceptions regarding IFRS. Although research on IFRS began in the late 90s, stakeholders’ perceptions were considered for research from 2005 onward due to the adoption of IFRS in 25 countries of Europe on January 1, 2005. However, as the time lapsed, research in this domain grew exponentially reaching new peaks, especially in 2013, 2016 and 2018. This growth can be attributed to the increasing number of jurisdictions agreeing to adopt or converge to IFRS. Contemporary studies are found to focus more on perceptions, emerging economies, fair value etc.

**Fig. 4:**
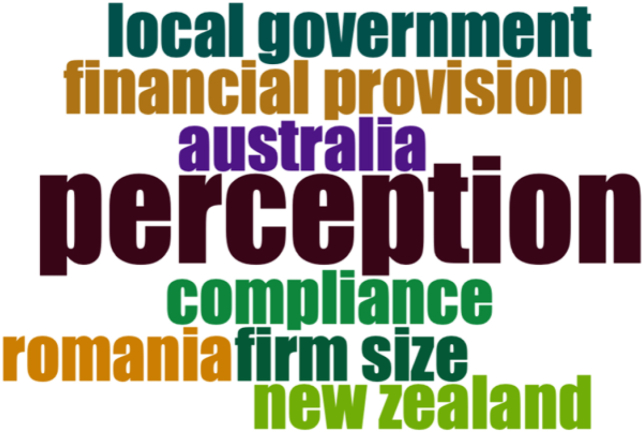
Keyword analysis.

### 4.3 Analysing authors, keywords and sources

There are in total 259 authors who contributed to 106 journal articles. These authors have an average local citation of 2.92. Sydney J. Gray and Mahesh Joshi emerge to have the highest h-index. The highest number of these articles bear the affiliation of RMIT University. They have mainly used keywords such as perception, compliance, firm size, local government, provision etc (see Fig. 4).

**Fig. 5:**
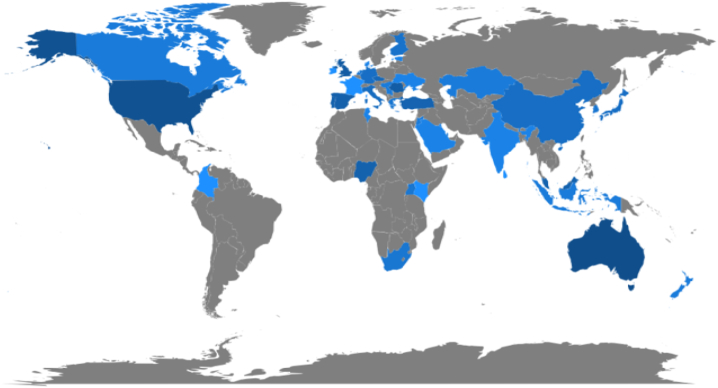
Countries with scientific production in the area.

The documents are distributed equally among many journals such as the Asian Review of Accounting, Journal of Applied Accounting Research, Australian Accounting Review and Journal of Financial Reporting and Accounting which are pioneering the advancement in this field (Table 3). So, 106 articles are spread across 66 sources. Therefore, future researchers can contribute to this domain. These journals have an average of 43 citations with Australian Accounting Review having the *highest h-index.*

**Table 3:** Categorization of the literature on the basis of publication source.

Sources	*n*	Sources	*n*
ASIAN REVIEW OF ACCOUNTING	5	AFRICAN JOURNAL OF ECONOMIC AND MANAGEMENT STUDIES	1
JOURNAL OF APPLIED ACCOUNTING RESEARCH	5	AFRO-ASIAN JOURNAL OF FINANCE AND ACCOUNTING	1
AUSTRALIAN ACCOUNTING REVIEW	4	COGENT ECONOMICS AND FINANCE	1
JOURNAL OF FINANCIAL REPORTING AND ACCOUNTING	4	CONTADURIA Y ADMINISTRACION	1
ABACUS	3	CORPORATE GOVERNANCE (BINGLEY)	1
ACCOUNTING FORUM	3	CRITICAL PERSPECTIVES ON ACCOUNTING	1
ACCOUNTING IN EUROPE	3	ECONOMIC ANNALS-XXI	1
CORPORATE OWNERSHIP AND CONTROL	3	ESTUDIOS DE ECONOMIA	1
JOURNAL OF INTERNATIONAL ACCOUNTING, AUDITING AND TAXATION	3	EUROPEAN ACCOUNTING REVIEW	1
RESEARCH IN ACCOUNTING REGULATION	3	GLOBAL FINANCE JOURNAL	1
ACCOUNTING EDUCATION	2	INTERNATIONAL BUSINESS REVIEW	1
ACCOUNTING HORIZONS	2	INTERNATIONAL JOURNAL OF ACCOUNTING	1
ACCOUNTING RESEARCH JOURNAL	2	INTERNATIONAL JOURNAL OF ACCOUNTING, AUDITING AND PERFORMANCE EVALUATION	1
EUROPEAN RESEARCH STUDIES JOURNAL	2	INTERNATIONAL JOURNAL OF DIGITAL ACCOUNTING RESEARCH	1
INTERNATIONAL JOURNAL OF ACCOUNTING AND INFORMATION MANAGEMENT	2	INTERNATIONAL JOURNAL OF ISLAMIC AND MIDDLE EASTERN FINANCE AND MANAGEMENT	1
INTERNATIONAL JOURNAL OF ECONOMICS AND FINANCIAL ISSUES	2	INTERNATIONAL JOURNAL OF LAW AND MANAGEMENT	1
INVESTMENT MANAGEMENT AND FINANCIAL INNOVATIONS	2	INTERNATIONAL JOURNAL OF MANAGERIAL AND FINANCIAL ACCOUNTING	1
JOURNAL OF ACCOUNTING, AUDITING AND FINANCE	2	INTERNATIONAL JOURNAL OF MANAGERIAL FINANCE	1
JOURNAL OF INTERNATIONAL FINANCIAL MANAGEMENT AND ACCOUNTING	2	INTERNATIONAL JOURNAL OF REVENUE MANAGEMENT	1
MANAGERIAL AUDITING JOURNAL	2	ISSUES IN ACCOUNTING EDUCATION	1
MEDITARI ACCOUNTANCY RESEARCH	2	JOURNAL FOR GLOBAL BUSINESS ADVANCEMENT	1
MEDITERRANEAN JOURNAL OF SOCIAL SCIENCES	2	JOURNAL OF ACCOUNTING AND ORGANIZATIONAL CHANGE	1
PROBLEMS AND PERSPECTIVES IN MANAGEMENT	2	JOURNAL OF APPLIED ECONOMIC SCIENCES	1
WSEAS TRANSACTIONS ON BUSINESS AND ECONOMICS	2	JOURNAL OF ECONOMICS, FINANCE AND ADMINISTRATIVE SCIENCE	1
ACADEMY OF ACCOUNTING AND FINANCIAL STUDIES JOURNAL	1	JOURNAL OF FINANCIAL REGULATION AND COMPLIANCE	1
ACADEMY OF STRATEGIC MANAGEMENT JOURNAL	1	JOURNAL OF ISLAMIC ACCOUNTING AND BUSINESS RESEARCH	1
ACCOUNTING AND BUSINESS RESEARCH	1	JOURNAL OF PROPERTY INVESTMENT AND FINANCE	1
ACCOUNTING REVIEW	1	LOCAL GOVERNMENT STUDIES	1
ACCOUNTING, AUDITING AND ACCOUNTABILITY JOURNAL	1	PACIFIC ACCOUNTING REVIEW	1
ACCOUNTING, ORGANIZATIONS AND SOCIETY	1	POLISH JOURNAL OF MANAGEMENT STUDIES	1
ADVANCED SCIENCE LETTERS	1	REVIEW OF ACCOUNTING AND FINANCE	1
ADVANCES IN ACCOUNTING	1	REVISTA BRASILEIRA DE GESTAO DE NEGOCIOS	1
AFRICAN JOURNAL OF BUSINESS AND ECONOMIC RESEARCH	1	SUSTAINABILITY (SWITZERLAND)	1

### 4.4 Contribution by countries

**Fig. 6:**
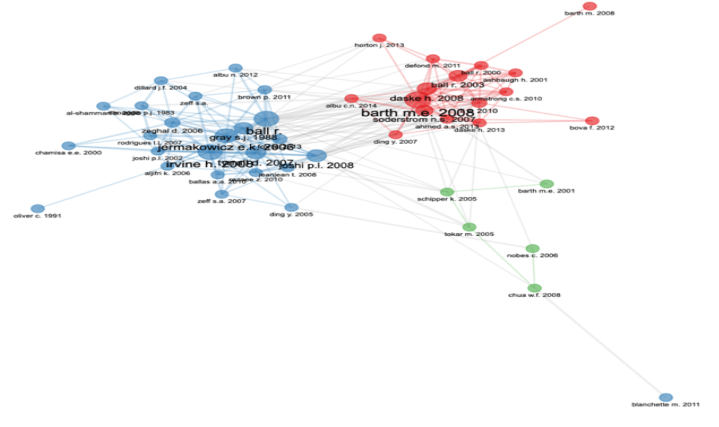
Author collaborations.

This analysis represents 41 countries that have been identified as having contributed to the area (Fig. 5). Ideally, the majority of the contribution should be coming out of the countries that are early adopters of IFRS. They have adopted the standards in 2005. These countries consist of Australia, the European Union (26 countries, including the UK) and Canada. However, the current bibliometric analysis found that the top 100 authors hail from Australia, the USA, the UK and Romania. The top countries with the highest citations include Germany, Australia, the UK and Romania implying that the German authors have far more reach and influence on budding as well as the contemporary researchers and other stakeholders than authors in any other nation. Surprisingly there were only two scientific productions from India implying the need for research about the perceptions of stakeholders in the convergence of IFRS in India. However, in the case of author collaboration with the authors from other countries, Australia ranked first, the USA secured second rank while the UK and Romania perched at third. Unfortunately, India's authors are nowhere to be seen having any foreign collaborations on the subject. In addition, other countries such as the Czech Republic, South Africa and Turkey had average contributions in the area. Fig. 6 below shows that Mary E. Barth, Jermakowicz, Irvine and Prof. Ray Ball emerge as the most collaborated authors.

From a continental analytical point of view, it is observed that Europe contributed the most followed by Oceania and North America. However, many African countries have made absolutely nothing of note. This can be linked to the countries' respective economic and demographic development. This research also reveals the *North-South gap* which is another intriguing finding. It has been found that nations that are north of the equator make greater contributions than nations south of the equator.

So, in this attempt at the bibliometric analysis for selected 106 scholarly articles, the results show that there are at least 14 citations on average for each of these articles. The peak of scientific research in the perceptional approach reached in the years 2013, 2016 and 2018. The authors who contributed majorly to the area are Sydney J. Gray and Mahesh Joshi. The 259 authors who contributed had a citation of around 3. These 106 articles are published in 66 journals with the highest number in the Asian Review of Accounting and the Journal of Applied Accounting Research. Amongst these 66 sources, the Australian Accounting Review has the highest *h-index*. The top 100 authors from this review emerged from Australia, the USA, the UK and Romania. However, Germany emerged as the country bringing the highest citations implying a foresighted and influential research community than in any other nation. Australia emerged as having the biggest collaboration network with authors from other countries. Mary E. Barth, Jermakowicz, Irvine and Prof. Ray Ball emerged as the most collaborated authors.

Unfortunately, only two papers from India were identified as quality publications on perceptional approach to be included in this review with Indian authors nowhere to be seen having any foreign collaborations on the subject opening the area for further research. It also highlights the need for more Indian researchers to work in the area.

## 5 Limitations and recommendations for future research

The present study conveys various concerns regarding IFRS implementation across various jurisdictions. Amongst them, analyzing stakeholders' perceptions represents an utmost important issue as perceptions help to understand a change from an individual's perspective. Seeking perceptions in the case of IFRS implementation is anticipated to help identify the benefits accrued and challenges posed by the change to various stakeholders in the normal course of financial reporting. Preparers and users form two important sects of stakeholders. The systematic review as presented in this piece of work addresses the views of various stakeholders from both sects. Extant literature diverges on that view with some authors favoring future studies on the perceptions of reporting users and others on preparers. Literature by far has shown that the preparers have been affected the most due to IFRS implementation hence has been researched the most and so posed a limitation of reigning most of the literature and so this review does not consider much of other stakeholders. Reporting users have been omitted from various studies including institutional lenders [38], financial analysts, stock-brokers, investors and government officials [5,39,40]. As pronounced by FASB, the plight of the financial statements users: *“Users of financial statements are shadowy figures within the paragraphs of financial accounting standards. They haunt these texts, lurking off-page, until the FASB claims to speak for them. Then, they are ushered briefly onto the pages to serve as an additional justification for changing existing accounting requirements. Even though users are rarely presented as actively seeking information or as being consulted by the FASB, they act as another source of authority for the FASB in its standard-setting process”*. Since, few studies have pointed towards the lack of studies in specific categories of users which provides an opportunity for the budding researchers with larger data sets of respondents [41]. They could include investment management firms [3]; Bankers [42] investors and financial analysts [38,43,44], auditors [45]; Regulators [38,44]; institutional leaders [5] and accounting researchers [44]. This recollection of studies on perceptions, hence, indicates the need to highlight the importance of ‘preparers’ and ‘users’ perceptions in the times of harmonizing accounting practices across the globe. Apart from analyzing stakeholders' perceptions, the following are a few limitations and avenues for future research:

a. ***Exploring the developing contexts****:* The extant literature on stakeholders' perceptions majorly confines to the developed countries. So, the results of this review focus on stakeholders' perceptions from the developed jurisdictions. The majority of these jurisdictions are a part of Europe, being one of the biggest proponents of the movement which augments the scope of research in Asia and other emerging regions. Future studies in emerging economies may focus on the role of the external auditor [46], implementation decisions and research base for policy [47], technical and organizational activities engaged by companies in emerging economies [45] and, compliance by the state-owned companies [40]. To generalize the findings of the research, comparative studies can also be considered as an existing research gap [3] which can be used as a means for determining whether differing economic environments [48] and multicultural settings [49] affect the respondents' views of accounting information. A particular avenue for comparative studies could be achieved by comparing a particular set of stakeholders' perceptions between developed countries and developing countries [44]. India has been identified as one such developing context as highlighted previously in this paper.
b. ***Factors affecting the stakeholders' perceptions of IFRS implementation:*** Given the scarcity of literature reporting the factors affecting the perceptions of stakeholders in the implementation of IFRS in various jurisdictions, the current review doesn't consider various factors affecting the implementation of IFRS in various contexts. So, “An investigation is required to ascertain whether specific companies' characteristics, such as the firm's size or the firm's ownership structure, can explain the managers' perceptions regarding the costs and benefits related to the introduction of IFRS” [43,50]. To this end [9], proposed a model including individual, technical, situational and change process factors whereas [51] apart from developing a model proposed other attributes as gaps in the literature to the like of institutional pressures, capital ownership, auditor types, professional status, and position that could be factored to drive the accountants' perception towards IFRS adoption. Both of these models can develop questionnaires for use in field interviews and survey-based research and serve as an important avenue for research to explore different and more developed instruments [52].
c. ***Empirical evidence of IFRS implementation****:* Due to the recent emergence of IFRS in many jurisdictions especially in Asia and Africa, there is a dearth of literature on empirical evidence due to the non-availability of data. Thus, this review doesn't include studies reporting stakeholders' perceptions of the empirical evidence. Hence, future reviews may include such studies. Apart from the avenues listed above, many studies have pointed out the lack of a priori evidence of the empirical effects of IFRS adoption. The future empirical research may provide evidence about the disclosure quality [53]; benefits, costs and effects of changing national accounting regulations/standards [54]; causes and consequences of various standards [[55], [56], [57]]; Capital market effects [58]; Earnings Management [46,59]; and compliance costs and its variable impact [60]. Methodological variation can also be considered in future research to provide a holistic view of the issue of IFRS adoption to improve the effective response rate [49]. This can be achieved in collaboration with authors from other areas of research or maybe different regions. Varied statistical and analytical methodologies like factor analysis (EFA/CFA) and structural equation modelling (SEM) [38] or the mix of qualitative and quantitative methods can be employed to draw more informative conclusions regarding the perception approach and the impact of IFRS adoption [5,60].
d. ***IFRS training and education****:* Many studies in the extant literature have highlighted the need for training and educating various stakeholders on the issue. However, there has been a lack of studies testifying stakeholders' perceptions on the same. So, the present review lacks analysis in this parlance. Nonetheless, preparing future accountants for IFRS implementation provides a futuristic gap for research. Assessing faculty members' beliefs about assessing students learning, and critical thinking abilities following the IFRS education [61]; continuing education of professionals [62]; IFRS teaching methods [63] specialized accounting transactions and unique organizations [47] and IFRS training [10,64] will add value to the IFRS literature.

## 6 Policy issues and implications

The objective of this paper is to present and classify the reviewed studies on IFRS adoption. This piece of literature may serve as a ready reckoner for the summary of existing and potential researchers and educators who wish to work in this area. This systematic review of the literature highlights the important areas of research that are useful to various stakeholders in IFRS implementation that have attracted the attention of the research community across jurisdictions. The database thus developed as part of this work highlights the preferred and weak areas of IFRS implementation. Indeed, budding researchers may focus on the areas that have received limited attention. The perceptions that affect the stakeholders on IFRS adoption are heavily endorsed by eminent researchers in the literature. Their findings have reasserted the importance of perceptions in decision-making. The current review provides readers with an understanding of the evolution of international financial reporting standards and its scholarly research. The educators can thus teach and update the students with the recent developments in the area. This review will further help educators in synthesising the concept, issues and trends in stakeholders’ perceptions regarding the implementation of IFRS across the globe.

In the development process of the accounting standards, the governments and other regulatory bodies need to keep abreast with the views of various stakeholders. So, these observations will guide further investigations not only to the stakeholders such as preparers (accountants) and users other than academicians and researchers but also to the policymakers, and regulatory and standard-setting bodies of the countries contemplating implementing IFRS in their jurisdictions.

## 7 Conclusion

This paper has reviewed the research literature on IFRS implementation in various contextual settings seeking perceptions of various stakeholder groups. The paper inferred that the existing studies vary in conclusions regarding the adoption of IFRS for various jurisdictions. The papers about the subject of this review depict a high average of citations per document which suggests an accelerated and exponential growth of academic articles in the area especially in 2013, 2016 and 2018 when the majority of jurisdictions agreed to adopt or converge to IFRS. These studies suggest that participants in some of the contexts mainly Asia-Oceania are very positive about adoption in the region. They perceive more benefits in the implementation of IFRS in their jurisdiction than costs, believing the net effect will be positive. However, in the other jurisdictions mainly Europe, the stakeholders perceive lesser benefits than costs. So, the perceptions may vary from region to region subject to many factors including the cultural setting of the context, legal orientation, support from the regulatory bodies and the firm-specific characteristics among many others.

Among the authors, Prof. Sydney J. Gray and Prof. Mahesh Joshi emerge to have the highest *h-index* having pioneering works in perception, compliance, firm size, local government, provision etc. in international journals such as Asian Review of Accounting, Journal of Applied Accounting Research, Australian Accounting Review and Journal of Financial Reporting and Accounting. While the top 100 authors come from Australia, the USA, the UK and Romania. After the detailed analysis of the studies reviewed it may be finally concluded that the adoption of IFRS may be directly or through a convergence process will be in the interest of world economies as it is going to create a new universally acceptable discipline of accounting called as ‘World Accounting’. Initially, there might be some challenges in moving towards IFRS but the overall benefits are perceived to be quite positive and remarkable. The implications of this study are projected towards bussing researchers, educators and policymakers. It further lists the limitations and provides avenues for future research. Similar studies may adopt a more comprehensive approach towards empirical evidence from developing nations which will strengthen the scope of the study.

## Data availability statement

No quantitative data was used for the research described in this article.

## CRediT authorship contribution statement

**Sakshi Bathla:** Methodology, Data curation. **Anil K. Sharma:** Conceptualization. **Vinay Kandpal:** Writing – review & editing, Investigation, Formal analysis.

## Declaration of competing interest

The authors declare that they have no known competing financial interests or personal relationships that could have appeared to influence the work reported in this paper.

## References

[1] Ball R. (Dec. 2006). International financial reporting standards (IFRS): pros and cons for investors. Account. Bus. Res..

[2] Yuan J., Jiang Y. (2008). Accounting information quality, free cash flow and overinvestment: a Chinese study. Bus. Rev..

[3] Georgiou G. (2010). The IASB standard-setting process: participation and perceptions of financial statement users. Br. Account. Rev..

[4] DeFelice A., Lamoreaux M.G. (2010). The SEC's IFRS work plan. J. Account..

[5] Phan D.H.T., Mascitelli B., Barut M. (Mar. 2014). Perceptions towards international financial reporting standards (IFRS): the case of vietnam. Glob. Rev. Account. Financ..

[6] Lourenço I.C., Sarquis R., Branco M.C., Pais C. (Jul. 2015). Extending the classification of European countries by their IFRS practices: a research note. Account. Eur..

[7] Lantin F., Tort É. (2015). Conséquences de l’adoption des IFRS sur l’information et les marchés financiers. Dix ans de littérature (2005-2014). Rev. française Gest..

[8] Covin T.J., Kilmann R.H. (Jun. 1990). Participant perceptions of positive and negative influences on large-scale change. Group Organ. Stud..

[9] Fontes A., Rodrigues L.L., Craig R. (2016). A theoretical model of stakeholder perceptions of a new financial reporting system. Account. Forum.

[10] Ben Abd El Afou R. (Jan. 2017). Knowledge of Islamic accounting among professionals: evidence from the Tunisian context. J. Islam. Account. Bus. Res..

[11] Armstrong C.S., Barth M.E., Jagolinzer A.D., Riedl E.J. (Sep. 2010). Market reaction to the adoption of IFRS in Europe. Account. Rev..

[12] Athma Prashanta R.N. (2013). Accounting standards in India : adoption of IFRS. J. Commer. Account. Res..

[13] Ray S. (2012). Indian GAAP and its convergence to IFRS: empirical evidence from India. Adv. Appl. Econ. Financ..

[14] Pacter P. (2017). Global reach of IFRS is expanding. CPA Journal, New York.

[15] Barth M.E., Clinch G., Shibano T. (1999). International accounting harmonization and global equity markets. J. Account. Econ..

[16] Ashbaugh H., Pincus M. (2001). No title. J. Account. Res..

[17] Barth M.E., Landsman W.R., Lang M.H. (Sep. 2008). International accounting standards and accounting quality. J. Account. Res..

[18] Jeanjean T., Stolowy H. (2008). Do accounting standards matter? An exploratory analysis of earnings management before and after IFRS adoption. J. Account. Publ. Pol..

[19] Ahmed A.S., Neel M., Wang D. (Dec. 2013). Does mandatory adoption of IFRS improve accounting quality? Preliminary evidence. Contemp. Account. Res..

[20] Barth M.E., Landsman W.R., Young D., Zhuang Z. (2014). Relevance of differences between net income based on IFRS and domestic standards for European firms. J. Bus. Financ. Account..

[21] Chen G., Kim K.A., Nofsinger J.R., Rui O.M. (Oct. 2007). Trading performance, disposition effect, overconfidence, representativeness bias, and experience of emerging market investors. J. Behav. Decis. Mak..

[22] Brüggemann U., Hitz J.-M., Sellhorn T. (May 2013). Intended and unintended consequences of mandatory IFRS adoption: a review of extant evidence and suggestions for future research. Eur. Account. Rev..

[23] Aisbitt S. (Oct. 2006). Assessing the effect of the transition to IFRS on equity: the case of the FTSE 100. Account. Eur..

[24] Sunder S. (2010). Adverse effects of uniform written reporting standards on accounting practice, education, and research. J. Account. Publ. Pol..

[25] Navarro-García J.C., Bastida F. (2010). An empirical insight on Spanish listed companies' perceptions of International Financial Reporting Standards. J. Int. Accounting, Audit. Tax..

[26] Schipper K. (Jan. 2005). The introduction of international accounting standards in Europe: implications for international convergence. Eur. Account. Rev..

[27] Patro A., Gupta V.K. (2012). Adoption of international financial reporting standards (IFRS) in accounting curriculum in India-an empirical study. Procedia Econ. Finance.

[28] Donnelly S. (Mar. 2007). The international accounting standards board. New Polit. Econ..

[29] Catty J.P. (2010). http://ndl.ethernet.edu.et/bitstream/123456789/27218/1/150.James%20P.%20Catty.pdf.

[30] Adhikari A., Bansal M., Kumar A. (2021). IFRS convergence and accounting quality: India a case study. J. Int. Accounting, Audit. Tax..

[31] Van der Meulen S., Gaeremynck A., Willekens M. (2007). Attribute differences between U.S. GAAP and IFRS earnings: an exploratory study. Int. J. Account..

[32] Agarwal R., Rastogi S., Mehrotra A. (2009). Customers' perspectives regarding e-banking in an emerging economy. J. Retail. Consum. Serv..

[33] Rookes P., Willson J. (2000).

[34] Burrell G., Morgan G. (1979).

[35] Tranfield D., Denyer D., Smart P. (Sep. 2003). Towards a methodology for developing evidence-informed management knowledge by means of systematic review. Br. J. Manag..

[36] Donthu N., Kumar S., Mukherjee D., Pandey N., Lim W.M. (2021). How to conduct a bibliometric analysis: an overview and guidelines. J. Bus. Res..

[37] Galletta S., Mazzù S., Naciti V. (2022). A bibliometric analysis of ESG performance in the banking industry: from the current status to future directions. Res. Int. Bus. Finance.

[38] Joshi M., Yapa P.W.S., Kraal D. (Jan. 2016). IFRS adoption in ASEAN countries. Int. J. Manag. Finance.

[39] McEnroe J.E., Sullivan M. (2013). An examination of the perceptions of auditors and chief financial officers regarding principles versus rules based accounting standards. Res. Account. Regul..

[40] Nurunnabi M. (Feb. 2017). Auditors' perceptions of the implementation of international financial reporting standards (IFRS) in a developing country. J. Account. Emerg. Econ..

[41] Wahyuni E.T., Lay P. (2010). Proceeding of Accounting National Symposium (Simposium Nasional Akuntansi) XIII.

[42] Albu N., Albu C.N., Bunea S., Girbina M.M. (2015). Accounting academia in emerging economies: evolutions and challenges. Int. J. Account. Inf. Manag..

[43] Naoum V., Sykianakis N. (2011). The perceptions of managers of Greek firms regarding the Costs and Benefits ensuing from the adoption of International Financial Reporting Standards in Greece. Int. J. Econ. Sci. Appl. Res..

[44] Yang H.H., Clark C., Wu C., Farley A. (2018). Insights from accounting practitioners on China's convergence with IFRS. Aust. Account. Rev..

[45] Obradović V., Čupić M., Dimitrijević D. (Mar. 2018). Application of international financial reporting standards in the transition economy of Serbia. Aust. Account. Rev..

[46] Barghathi Y., Collison D., Crawford L. (2017). Earnings management in Libyan commercial banks: perceptions of stakeholders. Int. J. Accounting, Audit. Perform. Eval..

[47] Joshi P.L., Bremser W.G., Al-Ajmi J. (2008). Perceptions of accounting professionals in the adoption and implementation of a single set of global accounting standards: evidence from Bahrain. Adv. Account..

[48] Benetti C. (2011). https://teses.usp.br/teses/disponiveis/12/12136/tde-01092011-184005/publico/CristianeBenetti.pdf.

[49] Sugahara S. (Sep. 2013). Japanese accounting academics' perceptions on the global convergence of accounting education in Japan. Asian Rev. Account..

[50] Papadatos K.P., Bellas A.P. (2011). Applying IFRS mandatory: evidence from Greek listed companies. Eur. Res. Stud. J..

[51] Phan D., Joshi M., Mascitelli B. (Jan. 2018). What influences the willingness of Vietnamese accountants to adopt International Financial Reporting Standards (IFRS) by 2025?. Asian Rev. Account..

[52] Holtzblatt M., Tschakert N., Abu Khadra H. (Aug. 2012). Teaching IFRS with online videos and webcasts: resources, analysis and guidance. Adv. Account. Educ..

[53] Daske H., Gebhardt G. (Sep. 2006). International financial reporting standards and experts' perceptions of disclosure quality. Abacus.

[54] Albu C., Albu N., Alexander D. (Apr. 2013). The true and fair view concept in Romania: a case study of concept transferability. Account. Cent. East. Eur..

[55] Pajunen K., Saastamoinen J. (Mar. 2013). Do auditors perceive that there exists earnings management in goodwill accounting under IFRS?: Finnish evidence. Manag. Audit J..

[56] Mardini G., Crawford L., Power D. (May 2015). Perceptions of external auditors, preparers and users of financial statements about the adoption of IFRS 8: evidence from Jordan. J. Appl. Account. Res..

[57] Mazzi F., Liberatore G., Tsalavoutas I. (Sep. 2016). Insights on CFOs' perceptions about impairment testing under IAS 36. Account. Eur..

[58] Nijam H.M. (Jan. 2016). Impact of IFRS adoption in Sri Lanka: an evaluation of financial reporters' perception. Int. J. Manag. Financ. Account..

[59] Damak-Ayadi S., Bensadon D., Praquin N. (2016). IFRS in a Global World: International and Critical Perspectives on Accounting.

[60] Pawsey N.L. (2017). IFRS adoption: a costly change that keeps on costing. Account. Forum.

[61] Zhu H., Rich K.T., Michenzi A.R., Cherubini J. (Nov. 2011). User-oriented IFRS education in introductory accounting at U.S. Academic institutions: current status and influencing factors. Issues Account. Educ..

[62] Morais M., Clea M. (2015). Standardization of Financial Reporting and Accounting in Latin American Countries.

[63] De Souza Costa P., De Sousa Gomes G., Braunbeck G.O., Santana M.E.G. (2018). A safari in Brazil: evidence regarding the framework-based approach to teaching. Rev. Contab. e Financ..

[64] Chukwunedu O., Tauringana V. (2012). From SAS to IFRS- : an investigation of Nigeria transition road map implementation problems. Res. Account. Emerg. Econ..

